# Apoptotic and predictive factors by Bax, Caspases 3/9, Bcl-2, p53 and Ki-67 in prostate cancer after 12 Gy single-dose

**DOI:** 10.1038/s41598-020-64062-9

**Published:** 2020-04-27

**Authors:** Carla Pisani, Martina Ramella, Renzo Boldorini, Gianfranco Loi, Michele Billia, Francesca Boccafoschi, Alessandro Volpe, Marco Krengli

**Affiliations:** 1 Department of Translational Medicine, University of Piemonte Orientale (UPO), via Solaroli 17, 28100 Novara, Italy; 2 Division of Radiation Oncology, University Hospital “Maggiore della Carità”, corso Mazzini 18, 28100 Novara, Italy; 3 Department of Health Science, University of Piemonte Orientale (UPO), via Solaroli 17, 28100 Novara, Italy; 4 Division of Pathology, University of Piemonte Orientale (UPO), via Solaroli 17, 28100 Novara, Italy; 5 Medical Physics, University Hospital “Maggiore della Carità”, corso Mazzini 18, 28100 Novara, Italy; 6 Division of Urology, University Hospital “Maggiore della Carità”, corso Mazzini 18, 28100 Novara, Italy

**Keywords:** Prostate cancer, Prostate cancer

## Abstract

Radio-induced apoptosis is mediated by the activation of tumor protein p53, Bax and caspases. The purpose of this study was to investigate the early activation of this pathway in men receiving *in vivo* irradiation immediately before radical prostatectomy for locally advanced prostate cancer. We also investigated cell proliferation index (Ki-67), proto-oncogene (p53) and anti-apoptotic protein (Bcl-2) levels as potential predictive factors. We selected a homogeneous sample of 20 patients with locally advanced prostate cancer and candidate to radical prostatectomy. To assess the apoptotic pathways, Bax, is studied through immunofluorescence assay, before and after 12 Gy single dose intraoperative radiotherapy (IORT) to the prostate, on bioptic samples and on surgical specimens. Moreover, before and after IORT, Bcl-2, p53, and Ki-67 were also detected through immunohistochemistry. A count of positive Bax spots for immunofluorescence was performed on tumor cells, prostatic intraepithelial neoplasia (PIN), and healthy tissue areas before and after IORT. We also analyzed Caspases 3 and 9 expressions after IORT. Before IORT, Bcl-2 mean value in neoplastic cells was 2.23% ± 1.95, mean Ki-67 in neoplastic area was 4.5% ± 3.8, and p53 was 22.5% ± 6.8. After IORT, Bcl-2 mean value in neoplastic cells was 8.85 ± 8.92%, Ki-67 in neoplastic area was 7.8 ± 6.09%, and p53 was 24.9 ± 26.4%. After the irradiation, healthy areas expressed significantly lower levels of Bax (2.81 ± 1.69%) with respect to neoplastic cells (p < 0.0001), while in PIN areas, Bax positive cells were significantly more present than in neoplastic areas (p = 0.0001). At statistical analysis, it was observed that cancer cells with Ki-67 ≥ 8% had a trend toward greater expression of Bax (p = 0.0641). We observed an increase of Bcl-2 expression after IORT in neoplastic areas (p = 0.0041). Biopsy specimens with p53 ≥ 18% and Ki-67 ≥ 8% had worse post-operative staging with extracapsular invasion (p = 0.04 for both parameters) and nodal positivity (p = 0.04 for p53 and p = 0.0001 at pathology for ki-67). No correlation between IORT and Caspases activation was noted. In conclusion, after 12 Gy IORT, Bax was overexpressed in tumor and PIN cells. Pre-operative Ki-67 and p53 definition could be used in future studies to predict patients with worse pathological stage, while Bcl-2 activation after IORT might be a predictive factor for loco-regional failure.

## Introduction

Intraoperative radiotherapy (IORT) is the ultimate expression of a dose-intensification treatment modality, with a high irradiation dose delivered during a surgical procedure. The rationale of hypofractionation and dose-intensification schemes of radiotherapy of prostate cancer is based on the particularly high level of sensitivity of prostate cancer cells to fraction size radiotherapy^1^.

The IORT technique was described in a previous study from our institution^2^.

Radiobiological studies suggest that the use of a high single dose of radiations might intensify treatment effectiveness by increasing the radio-induced intracellular death processes^3^. Of note, some Authors observed that doses greater than 10 Gy may act through permeability alterations on endothelial cells, most likely causing apoptosis by caspases activation^4^. Caspases could be activated in 3 pathways: the mitochondrial pathway, the extrinsic, and the intrinsic pathway of the endoplasmic reticulum.

Radiation induced damages, such as DNA injury, hypoxia, intracytoplasmic hypercalcemia, oxidative stress, could trigger the intrinsic pathway, which is the objective of the current study. Regardless of the stimuli inducing the apoptotic cascade, an increasing mitochondrial permeability, with subsequent release of pro-apoptotic molecules such as cytochrome c, will happen. This pathway is closely linked to a group of proteins belonging to the Bcl-2 family, named from the BCL-2 gene. There are two main groups of proteins belonging to the Bcl-2 family: pro-apoptotic proteins (Bax family) and anti-apoptotic (Bcl-2). Both intrinsic and extrinsic pathways converge on the common pathway and on the activation of caspase-3, that is the protein activating the nuclear damage.

Radiations cause a series of damage to cells and DNA, and radio-induced apoptosis is intermediated by the activation of p53, Bax and subsequent activation of caspases^5^. Cancer cells usually acquire auto-survival mechanisms and are resistant to apoptotic death, albeit there is no solid evidence describing the modalities of radio-induced apoptosis in prostate adenocarcinoma cells.

The purpose of this study was to investigate the early activation pathways of radio-induced apoptosis in radical prostatectomy and ultrasound-guided prostate biopsy specimens from men receiving IORT followed by radical prostatectomy for locally advanced prostate cancer. We assessed cell proliferation index (Ki-67), proto-oncogene (p53) and anti-apoptotic protein (Bcl-2) levels in different irradiated tissues including prostate cancer, PIN, and benign cells. The IORT represents an *in vivo* modality of irradiation. We further conducted the assessment for prognosticators of disease progression by analyzing the relation between molecular data and clinical and pathological features. These biological factors were correlated with postoperative pathological staging and biochemical local control considering a prostatic specific antigen (PSA) values higher than 0.2 ng/ml for tumor recurrence.

## Materials and Methods

We selected 20 men from a cohort of 132 patients treated by IORT, followed by radical prostatectomy and lymph node dissection for non-metastatic hormone-sensitive intermediate-high risk prostatic carcinoma as described in a previous article^2^.

Case selection was performed upon the completeness of parameters to be investigated in the biopsy and in the surgical specimen, and upon the length of follow-up.

IORT was delivered by a dedicated linear accelerator (Mobetron, Intraop, Sunnyvale, CA, USA) using electron beams of 9–12 MeV to a total dose of 12 Gy. The dose was prescribed to the 90% isodose covering the tumor volume and the surrounding healthy tissue, including PIN, where biopsies had been performed.

### Ethics approval and consent to participate

Our local ethics committee, “Comitato Etico Interaziendale Novara – AASSLL BI, NO, VCO, AOU “Maggiore della Carità” di Novara”, ruled that no formal ethics approval was required in this particular case because all the analysis was performed on histological specimens with no changes in patients treatments.

The policy of our institution is to allow investigations on patients’ tissues for those who signed an informed consent for a surgical procedure.

As a matter of fact, the informed consent for any surgical procedure includes a sentence in this regard.

All patients received and signed a specific informed consent before IORT and surgery. The study was performed in accordance with the Declaration of Helsinki.

### Histological analyses on prostate samples

Prostatic specimens were sent immediately after the surgical removal sent to the Pathology Unit and fixed in 10% buffered formalin within 90 minutes (mean 80 minutes, SD: 74–90 minutes) from surgery and within 120 minutes (mean 102 minutes, SD: 95–120) from IORT procedure.

From paraffin embedded tissues, 3–5 μm-thick sections were cut with a microtome (Leica, mod. Histo Slide 2000R, Wetzlar, Germany). To study cell proliferation and cell cycle, immunohistochemistry with anti-Ki-67 (1:250, Ventana ® Medical Systems, Roche, Monza, Italy) and anti p-53 (1:250, Ventana® Medical Systems, Roche, Monza, Italy) antibodies was performed by using an automated immunostainer (Ventana, Roche, Monza, Italy).

For tissue immunofluorescence, rehydrated samples were incubated with the following antibodies: anti human cleaved caspase-3 (1:200; Cell Signaling Technology Inc., Pero, Italy), anti-human caspase-9 and anti-Bax (1:200; Cell Signaling Technology Inc., Pero, Italy).

Detection of specific antigens was achieved by incubating the slides with 10% normal goat serum (NGS; Vector Laboratories, Peterborough, UK)–phosphate-buffered saline (PBS), to reduce non-specific binding, then with the following primary antibodies in 5% NGS overnight at 4C in a humid chamber. Subsequently, they were incubated with a FITC-conjugated secondary antibody (1:500, Vector, CA, USA). Slides were then counterstained with 4′,6-diamidino-2-phenylindole (DAPI, 1 microg/ml, Sigma-Aldrich, Milan, Italy), mounted with a mounting medium for fluorescence (Vectashield; Vector Laboratories, Peterborough, UK). Images were processed using a Leica fluorescence microscope (DM2500 Leica, Wetzlar, Germany) equipped with a digital camera. The samples were then acquired with Pannoramic MIDI (3DHISTECH Ltd, Budapest, Hungary), and analyzed with Pannoramic Viewer software (3DHISTECH, Budapest, Hungary).

After immunofluorescent staining and acquisition, samples were opportunely treated and stained using hematoxylin and eosin.

The urologist mapped the whole prostate and the intraprostatic dominant lesion with ultrasound guided prostate biopsy, and the pathologist reconstructed the site of the same lesion and surrounding tissues in the surgical specimen to compare the expression of apoptotic factors in the corresponding areas. Two bioptic specimens in the dominant lesion were analyzed for the current study to consider inter-tumoral heterogeneity.

Bax, caspases 3 and 9 positivity were measured with 40x magnification, on two healthy tissue fields within the irradiated area, four PINs fields and four neoplastic fields. Laboratory analyses were performed by a PhD molecular biologist, supervised by an expert pathologist.

### Statistical analyses

The results were analyzed using GraphPad Prism 4 software (GraphPad Software Inc., La Jolla, CA, USA). The apoptotic values highlighted with Bax expression in neoplasia and PIN areas with healthy tissue values had compared each other in the biopsy and in the surgical specimens. Apoptosis late pathway was assessed by Caspases 3 and 9 which were analyzed in surgical specimens in tumor, PIN and healthy tissue within the irradiated area. Friedman ANOVA and Wilcoxon tests were used to assess the differences of Bax expression among the samples. Results with p-values <0.05 were considered significant. Aware of the limited sample in our study, we evaluated the values of p53, Ki-67 and Bcl-2 as prognostic factors of Bax with a descriptive statistic.

## Results

Characteristics of the patients including postoperative tumor staging are listed in Table 1. Median follow-up of the study cohort was 63.6 months ± 9 months. Fourteen out of 20 patients (70%) experienced biochemical failure and no patient developed distant metastases.

**Table 1:** Clinical and pathological features of the 20 patients in study.

Characteristics	Value and IQR
Mean age at diagnosis (SD)	65 years (52–74)
Mean performance status at diagnosis (SD)	90 (80–100)
Mean initial PSA (SD)	17 ng/ml (4.47–41)
Neoadjuvant hormonal therapy	0
Pathological stage	**Absolute #**
pT2c	2
pT3a	4
pT3b	12
pT4	2
pN0	15
pN1	5
Adjuvant external beam radiotherapy	18 patients

Bioptic specimens were withdrawn 32 days (mean 32 days, SD: 26–45) before surgery.

With p53 antibodies used in our study, higher p53 expression is related to the presence of a mutated protein isoform, being the wild type protein quickly eliminated by intracellular systems.

Specimens from prostate biopsies showed that prostate cancer cells had a Bcl-2 mean value of 2.2% ± 1.9, Ki-67 of 4.5% ± 3.8, and p53 of 22.5% ± 6.8.

Table 2 shows the results of Bax analysis on neoplastic, pre-neoplastic and healthy tissue areas. Table 3 shows the results of immunohistochemistry analysis, expressed as percentages of positivity of Ki-67, p53, and Bcl-2 in cancer cells following IORT. No statistical difference was observed in terms of Ki-67, p53, and Bcl-2 expression levels between normal and neoplastic cells (p > 0.05).

**Table 2:** Bax expression levels after and prior IORT: the first 3 columns show the percentage of Bax positivity in tumor, PIN and healthy tissue fields out of all cells (DAPI positive), while the last column shows the percentage of Bax cells positivity before IORT.

#case	Bax/DAPI (%) neoplasic fields	Bax/DAPI (%) preneoplasic fields	Bax/DAPI (%) healthy tissues fields	Bax/DAPI (%) Biopsy fields
#1	8.40	19.60	0.40	1.04
#2	8.81	21.09	4.84	3.40
#3	4.69	7.56	0.55	1.42
#4	6.74	17.80	3.10	1.16
#5	2.15	28.86	3.54	2.51
#6	17.02	24.42	4.41	0.46
#7	5.82	19.12	1.31	2.86
#8	2.50	34.73	2.17	1.91
#9	17.02	24.42	6.00	0.31
#10	8.38	17.67	2.57	0
#11	7.25	23.48	3.84	1.71
#12	12.08	10.85	0.41	1.12
#13	7.46	19.1	1.25	0.58
#14	8.42	31.56	1.98	0.96
#15	4.58	18.74	0.84	1.24
#16	3.21	21.48	2.74	0.98
#17	9.58	23.5	5.40	0
#18	6.47	9.15	2.96	0
#19	12.90	26.84	4.10	2.11
#20	7.25	23.90	3.84	1.90
Mean value ± standard deviation	8.04 ± 4.15	21.19 ± 6.90	2.81 ± 1.69	1.28 ± 0.96

**Table 3:** The expression of proteins under investigations (p53, Bcl-2, Ki67) in neoplastic fields after IORT.

#case	p53 (%)	Bcl-2 (%)	Ki67 (%)
#1	7.2	4.1	4.2
#2	19.3	<1	17.2
#3	<1	23.2	9.6
#4	<1	18.2	5.4
#5	41.1	2.3	9.4
#6	86.2	19.3	16.2
#7	<1	<1	7.1
#8	39.2	<1	2.4
#9	18.3	<1	<1
#10	7.3	17.5	7.4
#11	20.3	19.2	7.1
#12	7.2	<1	18.3
#13	22.2	4.3	<1
#14	28.2	28.5	6.3
#15	10.1	<1	8.2
#16	25.3	10.4	21.2
#17	94.3	7.1	7.1
#18	<1	3.2	2.3
#19	32.2	15.4	<1
#20	45.2	2.3	7.1
Mean value ± standard deviation	24.9 ± 26.4	8.8 ± 8.9	7.8 ± 6.1

**Figure 1:**
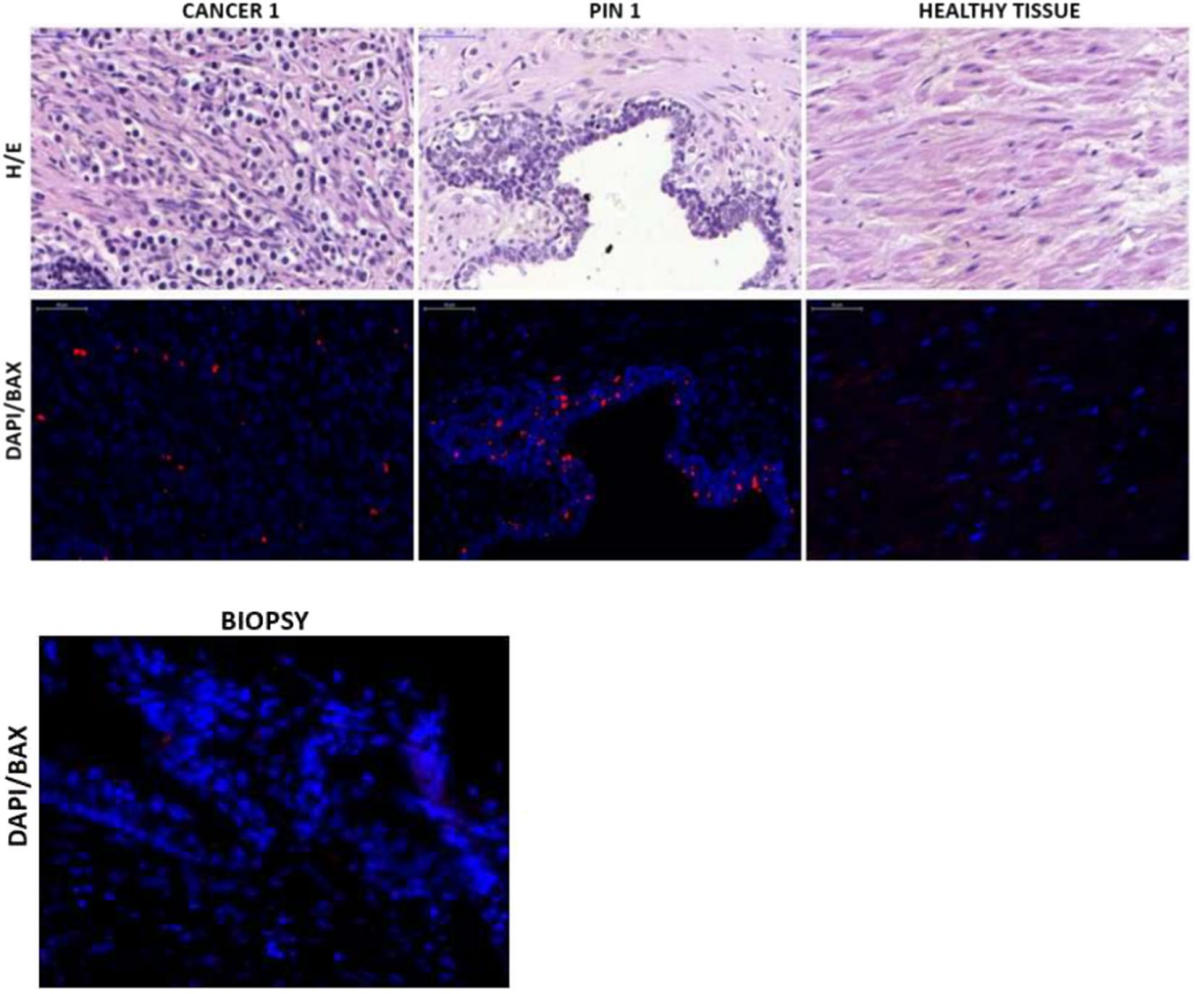
Hematoxylin/eosin (H/E) in surgical specimen and immunofluorescence fields for Bax (DAPI/BAX) (pt #9) in surgical and biopsy specimens. In blue all DAPI (4′,6-diamidino-2-phenylindole) positive cells (all nucleate cells), in red the cells that expressed Bax.

Figure 1 shows a neoplastic (cancer 1), a PIN (PIN 1), and a healthy tissue field in hematoxylin/eosin and immunofluorescence, and biopsy neoplastic fields in Bax immunofluorescence. There were significant differences in Bax expression among healthy tissue, PIN and cancer fields as resulted from Friedman ANOVA (p < 0.0001) comparing the irradiated samples. The pairwise Wilcoxon test showed that Bax was significantly overexpressed in neoplastic (p = 0.0001), PIN fields (p = 0.0001) and healthy cells after IORT (p = 0.003) compared to biopsy specimens before IORT.

We found a significantly increase of Bcl-2 expression after IORT in neoplastic areas (p = 0.0041). No differences were found in p53 and ki-67 expression before and after IORT in neoplastic cells.

From the multiple regression analysis, we did not find any correlation between p53, Bcl-2 and ki-67 expression and Bax activation after IORT.

Of note, we observed a significant overexpression of Bcl-2 on cancer cells following IORT (p = 0.004), while no differences were found in p53 and ki-67 expression prior and after IORT in neoplastic cells.

From the correlation between Ki-67, p53, and Bcl-2 values with the levels of expression of the Bax apoptotic protein. We observed that cancer cells receiving IORT had a greater trend towards apoptosis when Ki-67 levels were greater than 8.4% (p = 0.064). However, with multiple regression analysis, we did not find any correlation between p53, Bcl-2 and ki-67 expression and Bax activation after IORT.

Interestingly, we noted that patients harboring p53 levels >18% and ki-67 levels >8% on biopsy specimens had an increased likelihood to detect extracapsular invasion (p = 0.04 for both parameters) and nodal positivity (p = 0.042 for p53 and p = 0.0001 at pathology for ki-67). We chose the median value of 8% to discriminate patients with high and low proliferative index. p53 value of 18% was chosen according to values distribution in our sample because it represented the median one.

**Figure 2:**
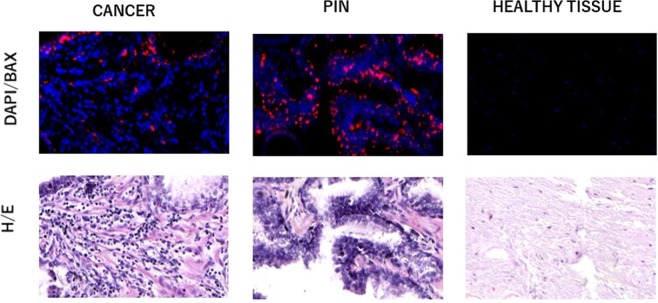
Hematoxylin/eosin (H/E) and immunofluorescence fields for Caspase 3 (DAPI/BAX) (pt #9) in surgical specimen. In blue all DAPI (4′,6-diamidino-2-phenylindole) positive cells (all nucleate cells), in red the cells that expressed Caspases 3.

Figure 2 show neoplastic (cancer 1), PIN (PIN 1) and healthy tissue field in the surgical specimen with hematoxylin/eosin staining and immunofluorescence for Caspases 3.

**Figure 3:**
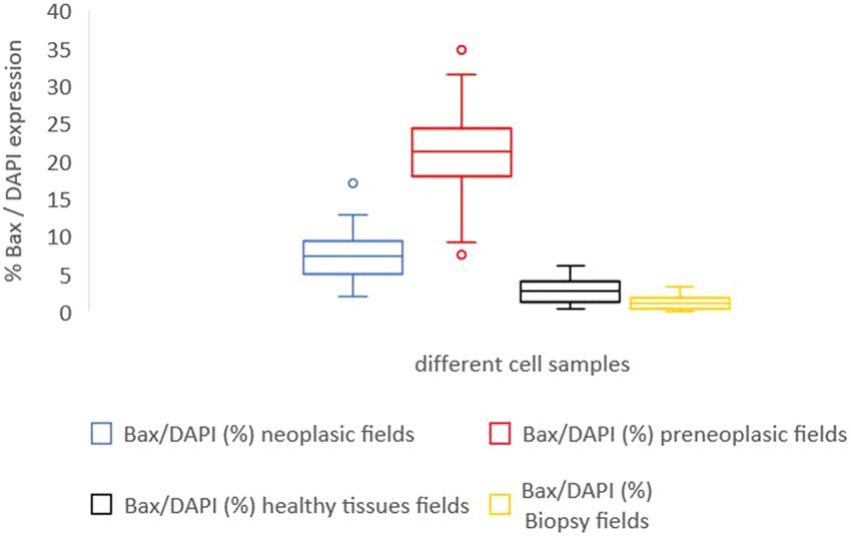
Box plot representation of Table 2 – Bax/DAPI expression in neoplastic (blue plot), preneoplastic (red plot), healthy tissue samples (black plot) after IORT and in bioptic specimen (yellow plot) before IORT.

**Figure 4:**
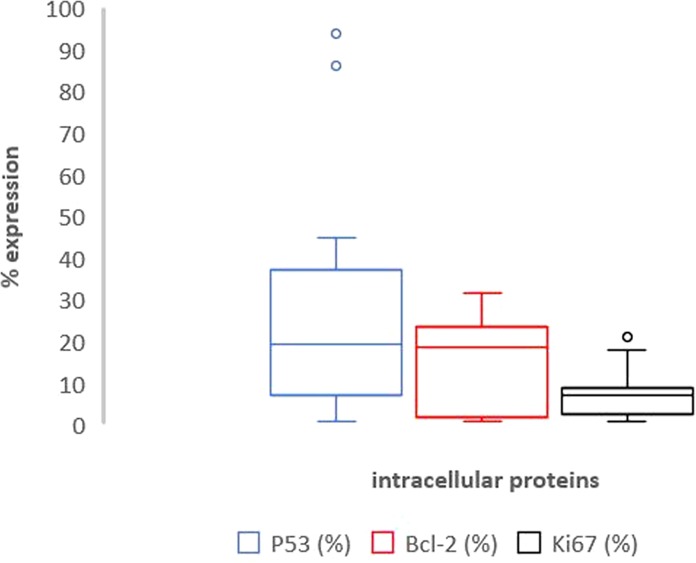
Box plot representation of Table 3 – p53 (blue plot), Bcl2 (red plot) and ki-67 (black plot) expression in neoplastic fields after IORT.

**Figure 5:**
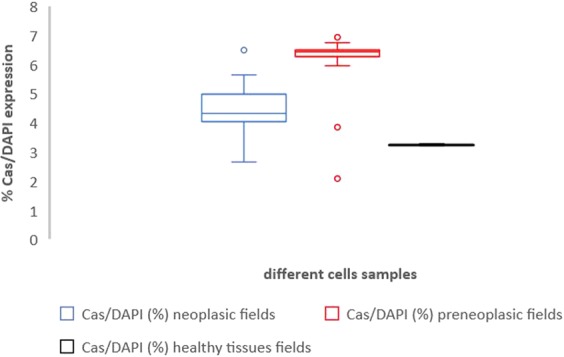
Box plot representation of Table 4 – Caspases/DAPI expression in neoplastic (blue plot), preneoplastic (red plot) and healthy tissue samples (black plot) after IORT.

After IORT, average Caspase 3 and 9 expressions were 4.32 ± 0.89 in cancer fields, 6.46 ± 1.70 in PIN areas, and 3.27 ± 0.02 in healthy tissue cells (Table 4). There were no significant differences of expression of such proteins among neoplastic, pre-neoplastic, and normal tissue cells (p > 0.05). As far as Bcl-2 values are concerned, we observed that patients with levels of Bcl-2 prior IORT higher than 9% had an increased risk of biochemical failure (p = 0.004). The 9% threshold was chosen since it represented the median value in our patient sample. In Figs. 3–5, and Table 5, we reported box plots and the results to summarize our findings.

**Table 4:** Caspases 3 and 9 expression levels out of all cells (DAPI positive) in neoplastic, preneoplastic and healthy tissue samples after IORT.

#case	Cas/DAPI (%) neoplasic fields	Cas/DAPI (%) preneoplasic fields	Cas/DAPI (%) healthy tissues fields
#1	4.12	6.49	3.24
#2	4.33	3.85	3.23
#3	6.49	11.85	3.28
#4	4.03	6.41	3.22
#5	4.98	6.44	3.26
#6	5.64	6.74	3.27
#7	4.12	6.11	3.27
#8	4.33	6.44	3.26
#9	2.66	2.1	3.29
#10	3.16	6.45	3.24
#11	4.31	6.72	3.28
#12	4.48	6.51	3.26
#13	5.01	6.43	3.24
#14	4.33	6.4	3.25
#15	3.64	6.25	3.27
#16	3.66	6.47	3.29
#17	5.64	5.98	3.26
#18	4.3	8.24	3.25
#19	4.79	6.45	3.25
#20	5.11	6.95	3.23
Mean value ± standard deviation	4.32 ± 0.89	6.46 ± 1.70	3.27 ± 0.02

**Table 5:** Summary of results differentiated by protein values (% mean value ± standard deviation) and study time.

Protein	% neoplasic fields after IORT	% preneoplastic after IORT	% healthy tissue fields after IORT	% before IORT (tumor area)
Bax	8.04 ± 4.15	21.19 ± 6.9	2.81 ± 1.69	1.28 ± 0.96
Caspases	4.32 ± 0.89	6.46 ± 1.70	3.27 ± 0.02	/
p53	24.9 ± 26.4	/	/	/
Bcl-2	8.85 ± 8.92	/	/	/
ki-67	7.8 ± 6.09	/	/	/

## Discussion

Patients with intermediate and high-risk prostatic cancer experience biochemical recurrence in 24–72% of cases after radical surgery or radiation^6^. Understanding the molecular pathways regulating apoptosis of prostate cancer cells due to hypo-fractionated radiotherapy is still a daunting task for physicians. There is very little evidence^7,8^ about the radiobiological effects on tissues of single-shot radiation suggesting a possible endothelial damage to peritumoral vessels with consequent hypoxia and cellular death.

The interest of studying biomolecular changes after IORT resides in the possibility to better understand mechanisms of cell death related to the use of extreme hypofractionation which is of increasing interest for external beam radiotherapy of prostate cancer. In this regard, IORT represents an ideal opportunity to investigate radiation related changes in tumor and healthy tissues just after irradiation and immediately before tissue withdrawn and pathology examination.

Some studies showed that hormonal therapy and a few chemotherapy drugs can induce apoptosis^9,10^. Due to this evidence we included in the study hormone-naïve patients.

We focused our analysis on the mechanisms related to the mitochondrial apoptotic pathway of cellular death following single-shot irradiation, evaluating the “*in vivo*” radio-induced damage received by tissues.

Prior to radiation, levels of Bax protein were significantly lower compared to PIN and neoplastic cells treated with IORT (p < 0.05). However, prior to a single-shot irradiation, neoplastic and pre-neoplastic cells do not express apoptosis proteins. This data suggests that IORT could be able to activate apoptosis in prostate cancer cells.

We observed that Bax protein is significantly increased in PIN cells (p < 0.0001) and in cancer cells (p = 0.006) following IORT. Interestingly, PIN areas appeared to be more sensitive to irradiation than normal prostatic tissue in our study population. No significant correlation was observed between Bax expression and PSA at diagnosis or Gleason Score at histology, and no correlation between IORT and Caspases activation was noted. Our data suggests that the activation of caspases occurs later than Bax pathway involvement. In this regard, we did not investigate caspase expression in the biopsy specimen since there was no activation of apoptosis, according to the negative Bax results. In 1995, Raffo *et al*. first demonstrated that Bcl-2 oncoprotein could protect prostate cancer cells from apoptotic stimuli^11^. Nowadays, there is evidence that proteins of the Bcl-2 family may play a role in the development of human malignancies and may act as key players in the process of programmed cell death.

Non-neoplastic prostate cells should express Bcl-2 levels of about 2–3%^12^. A recent review showed that Bcl-2 hyperexpression in tumor cells is associated with good prognosis in colorectal, breast, non-small cell, glioma, and gastric cancers. According to such review, measuring the levels of expression of Bcl-2 could be used to stratify patients and understand the response to active treatments^13^. Other “*in vitro*” studies demonstrated that Bcl-2 overexpression confers resistance to hormonal therapy among prostate cancer patients^14^. Our results are consistent with these literature data. We observed that increased expression of Bcl-2 following IORT in prostate cancer cells was associated with an increased risk of a local relapse. Based on our findings, it is reasonable to hypothesize that the expression of Bcl-2 after IORT may activate intracellular mechanisms leading to radio-resistance.

Several studies investigated the predictive and prognostic role of Bax and Bcl-2 family proteins^15,16^. Clinical data from RTOG 86–10 and RTOG 92–02 showed that only Bax expression at a normal level was associated with significantly more favorable outcome^17^. *In vitro* data showed conflicting results with studies without significant differences in the expression of p53, Bcl-2 and Bax 2 and 4 hours after 10 Gy into cell lines^18^ and studies showing that single shot irradiation could induce Bax-mediated cell death *in vitro*^19^. Our work seems to show that this process could happen “*in vivo*” as well.

To our knowledge our study is the first to describe that a single-shot irradiation may induce Bax-mediated cell death in patients receiving IORT, that represents an *in vivo* irradiation modality allowing for a rapid subsequent pathological examination of the irradiated tissue.

It has long been known that PIN areas are closely related to the presence of prostate cancer. By now, all literature data agree that neoplastic areas are related to intracellular mutations in pre-neoplastic areas. Recently, PIN morphological alterations have been shown to be associated with an increased replication index^20^. Xie *et al*. demonstrated in an animal model that pre-neoplastic cells with Bcl-2 hyperexpression have higher proliferative index, and increased expression of Bax. An increased apoptotic rate in high grade pre-neoplastic cells probably implicates that apoptosis may accelerate cellular turnover in premalignant lesions of the prostate. According to this animal model, the well differentiated neoplastic cells possibly developed a genetic profile of natural resistance against apoptotic stimuli^21^. We could hypothesize that PIN cells are most susceptible to irradiation, because they have already a high turnover.

To our knowledge, this is one of the first studies that showed that “*in vivo*” pre-neoplastic cells are more prone to apoptosis to single dose irradiation than neoplastic prostate cells. Interestingly, cancer cells present a significantly lower Bax positivity profile than PIN areas, most likely due to a relative radio-resistance induced by cancer transformation itself.

In some neoplasms, such as breast cancer, a correlation between Ki-67 value, and response to adjuvant treatments were observed. Of note the literature is poor when prostate cancer is concerned. Most likely, Ki-67 values in prostate carcinoma would be extremely heterogeneous as observed by Mesko *et al*. who reported values ranging between 1.1 and 10.1%^22^. Of note, Ki-67 is higher among patients with locally advanced prostate cancer. *In vitro*, it was observed that higher-proliferating cells were also those that tend to hyper-express apoptotic proteins after extracellular stimuli^12^.

In our sample, patients had a mean Ki-67 value of 7.8% ± 5.1%. We chose the median value of 8% to discriminate patients with high and low proliferative index. We observed that cells with Ki-67 > 8% had an increased trend towards apoptosis (p = 0.0641). Therefore, even *in vivo*, there could be an increased sensitivity to single shot irradiation with the increase of the proliferation index.

In our biopsy samples, higher proliferation index and higher p53 expression were associated with worse pathological tumor stage, higher incidence of extracapsular extension, and higher risk of nodal disease. Our data supports those from previous studies by Saidi *et al*.^23^ and Berlin *et al*.^24^. Based on our results, we could hypothesize that p53 protein and Ki-67 could be used as prognostic factors. This data may be of great interest in routine clinical practice as there is no current prognosticator of extra-prostatic extension of cancer.

In p53 mutated neoplastic cells, we observed a lower expression of Bax (p = 0.5), while there was a significant increase in expression of Bax in PIN areas (p = 0.04) and in healthy tissue areas (p = 0.02). In this regard, p53 responds to radiation-induced damage in several ways, such as inducing cell cycle arrest and activating apoptosis^25^. Some *in vitro* studies underlined that the activation of p53 protein increases the radio sensitivity of prostate cancer cells^26,27^. On the contrary, other studies concluded that p53 expression does not influence radiation sensitivity in prostate carcinoma^28,29^.

Our *in vivo* study seems to confirm, indeed, that neoplastic cells with mutation in p53 are less sensitive to apoptosis induced by single dose irradiation than healthy cells and surrounding PIN areas. On the contrary, non-mutated p53 cells (p53 < 18%) resulted more sensitive in tumor than in PIN and normal tissue cells. It can be reasonably hypothesized that in PIN and healthy cells p53 protein is still functioning and it is able to trigger the apoptosis after radio-induced damage.

We acknowledge that our study has limitations. This is a single center study based on small patient population. We are further conscious that in our study we did not investigate intracellular changing connected we hypoxia that could be matter of a further investigation. In the next future, we would like to study the expression of a transcription factor protein, hypoxia-inducible factor 1alpha (HIF-1alpha), to differentiate tissue changing related to surgery stress and IORT. However, the original design of our study, based on a translational research approach, has the strength to first report *in vivo* novel findings of molecular biology of mechanisms of apoptotic pathway in prostate cancer cells treated with single-dose radiotherapy.

## Conclusion

Our study showed that mitochondrial apoptosis and Bax pathway is activated in a few minutes after irradiation in prostate cancer cells following a single high dose radiation.

In our study, the cell death program was significantly activated among cancer cells and PIN tissues, whereas this result could not be observed in benign cells. These findings support the role of radiations as a precise carrier of a cell damage specifically directing towards cancer cells, while sparing benign tissues most likely due to the preservation of anti-apoptotic mechanisms.

From our analysis, it emerges that neoplastic cells with higher proliferating index are more responsive to radio-induced damage. On the other hand, higher Ki-67 and mutated p53 cells are predictive for higher pathological staging, extra-capsular extension, and nodal disease. Mutated p53 is also predictive for radio-resistance. We also noticed that pre-operative and post-operative Bcl-2 might predict biochemical failure. These elements, if confirmed in larger cohorts, could help to stratify patients in clinical studies and to select which patients could benefit the most from highly hypofractionated regimens possibly including intraoperative irradiation.

## Data Availability

Information on data supporting the results reported in the article can be found in a dataset of the University Hospital “Maggiore della Carità”, Novara, Italy.
